# Supplementary material for: Glycolysis Is an Intrinsic Factor for Optimal Replication of a Norovirus
Source: mBio. 2019 Mar 12;10(2):e02175-18. doi: 10.1128/mBio.02175-18 (PMC6414699; doi:10.1128/mBio.02175-18)
Supplement: TABLE S1 [file mBio.02175-18-st001.docx]

**Supplemental Table 1. Metabolomics results from MNV-1 infected RAW 264.7 cells.** Quantitative Univariate Analysis from Metaboanalyst (data normalized to protein content)

| **Name** | **Mean (SD) of MNV infected** | **Mean (SD) of mock lysate** | **p-value** | **q-value (FDR)** | **Fold Change** | **MNV infected/mock lysate** |
| --- | --- | --- | --- | --- | --- | --- |
| FBP | 163.340 (11.956) | 75.787 (29.029) | **0.0047** | **0.042** | 2.16 | Up |
| ATP | 1220.538 (55.783) | 790.018 (160.112) | **0.0072** | **0.042** | 1.54 | Up |
| MAL | 627.984 (80.416) | 356.468 (84.888) | **0.0079** | **0.042** | 1.76 | Up |
| 6PG | 6.951 (1.529) | 3.329 (0.887) | **0.0104** | **0.042** | 2.09 | Up |
| CIT/ICIT | 492.318 (52.808) | 297.288 (73.279) | **0.0117** | **0.042** | 1.66 | Up |
| 2PG/3PG | 34.697 (11.032) | 18.750 (3.474) | **0.0384** | 0.1151 | 1.85 | Up |
| NADP | 21.962 (2.042) | 15.515 (4.551) | 0.0745 | 0.1915 | 1.42 | nd |
| R5P_X5P | 22.289 (3.862) | 14.095 (6.787) | 0.1234 | 0.2607 | 1.58 | nd |
| SUC | 108.774 (15.413) | 71.642 (32.349) | 0.1304 | 0.2607 | 1.52 | nd |
| FAD | 2.985 (0.577) | 2.293 (0.539) | 0.1631 | 0.2886 | 1.3 | nd |
| F6P/G6P | 228.313 (50.092) | 170.785 (49.436) | 0.1901 | 0.2886 | 1.34 | nd |
| NAD | 103.051 (24.241) | 74.938 (24.692) | 0.1935 | 0.2886 | 1.38 | nd |
| NADH | 1.746 (0.815) | 7.863 (7.658) | 0.2084 | 0.2886 | -4.5 | nd |
| E4P | 631.375 (232.385) | 464.782 (184.768) | 0.3363 | 0.4324 | 1.36 | nd |
| AMP | 69.282 (32.265) | 54.214 (49.835) | 0.6703 | 0.8044 | 1.28 | nd |
| ADP | 131.762 (114.584) | 106.970 (72.364) | 0.7376 | 0.8178 | 1.23 | nd |
| S7P | 33.646 (16.551) | 29.858 (16.025) | 0.7723 | 0.8178 | 1.13 | nd |
| aCoA | 2.246 (0.232) | 2.163 (0.687) | 0.8517 | 0.8517 | 1.04 | nd |

**Abbreviations:** FBP = Fructose bisphosphate; ATP = Adenosine triphosphate; MAL = Malate; 6PG = 6-phosphogluconate; CIT/ICIT = Citrate / Isocitrate; 2PG/3PG = 2- and 3-Phosphoglycerate; NADP = Nicotinamide Adenine dinucleotide phosphate (oxidized); R5P_X5P = Ribose-5-phosphate / Xylulose-5-phosphate; SUC = Succinate; FAD = Flavin adenine dinucleotide; F6P/G6P = Fructose- and Glucose-6-phosphate; NAD = Nicotinamide adenine dinucleotide (oxidized); NADH = Nicotinamide adenine dinucleotide (reduced); E4P = Erythrose-4-phosphate; AMP = Adenine monophosphate; ADP = Adenine diphosphate; S7P = Sedoheptulose-7-phosphate; aCoA = Acetyl CoA

nd = not determined with confidence

Analysis = Student’s two-tailed t-test
