# Supplementary material for: Glycolysis Is an Intrinsic Factor for Optimal Replication of a Norovirus
Source: mBio. 2019 Mar 12;10(2):e02175-18. doi: 10.1128/mBio.02175-18 (PMC6414699; doi:10.1128/mBio.02175-18)
Supplement: TABLE S2 [file mBio.02175-18-st002.docx]

**Supplemental Table 2. Metabolomics results from MNV-1 infected RAW 264.7 cells. Semi-Quantitative Univariate Analysis from Metaboanalyst (data normalized to protein content)**

| **Name** | **Mean (SD) of MNV infected** | **Mean (SD) of mock lysate** | **p-value** | **q-value (FDR)** | **Fold Change** | **MNV infected/mock lysate** |
| --- | --- | --- | --- | --- | --- | --- |
| Hypoxanthine | 27994.000 (1020.516) | 17400.000 (2725.504) | **0.0015** | **0.0619** | 1.61 | Up |
| UTP | 92509.000 (5528.810) | 60449.000 (10406.285) | **0.005** | **0.0619** | 1.53 | Up |
| Glutathione-Reduced | 1198629.333 (58425.057) | 837300.000 (131516.213) | **0.0073** | **0.0619** | 1.43 | Up |
| Xanthine | 22481.000 (5858.513) | 5448.750 (5029.716) | **0.0089** | **0.0619** | 4.13 | Up |
| Methionine | 18937.667 (1964.049) | 13011.750 (1814.529) | **0.009** | **0.0619** | 1.46 | Up |
| Proline | 52479.667 (477.939) | 35823.250 (5597.532) | **0.0091** | **0.0619** | 1.46 | Up |
| Creatinine | 5108.667 (355.854) | 3530.000 (578.726) | **0.0092** | **0.0619** | 1.45 | Up |
| Glutamine | 1423075.333 (52858.822) | 919491.250 (222531.711) | **0.0132** | 0.077 | 1.55 | Up |
| IMP | 927.333 (83.050) | 620.250 (123.543) | **0.0142** | 0.077 | 1.5 | Up |
| UDP-D-glucose | 91909.667 (6194.269) | 62023.750 (13912.078) | **0.019** | 0.0884 | 1.48 | Up |
| Alanine | 45887.000 (4080.852) | 31595.500 (6825.651) | **0.0245** | 0.0884 | 1.45 | Up |
| Asparagine | 11445.333 (1496.898) | 7771.750 (1602.369) | **0.0274** | 0.0884 | 1.47 | Up |
| Tyrosine | 77226.667 (8578.783) | 57073.750 (8554.227) | **0.0274** | 0.0884 | 1.35 | Up |
| Phenylpyruvate | 2093.667 (264.530) | 1512.500 (237.911) | **0.0282** | 0.0884 | 1.38 | Up |
| Dihydroxy-acetone phosphate | 8452.333 (335.524) | 5578.250 (1571.700) | **0.0286** | 0.0884 | 1.52 | Up |
| 3-Phospho-serine | 11728.000 (1872.546) | 7792.500 (1569.319) | **0.0289** | 0.0884 | 1.51 | Up |
| Arginine | 43660.000 (3260.505) | 31607.500 (6201.255) | **0.0295** | 0.0884 | 1.38 | Up |
| Gluconate | 3247.333 (955.861) | 1713.500 (479.001) | **0.0366** | 0.0935 | 1.9 | Up |
| Glutamate | 1087814.000 (167819.574) | 760778.750 (142649.861) | **0.0382** | 0.0935 | 1.43 | Up |
| Ornithine | 34491.333 (3045.479) | 24714.500 (5398.408) | **0.0389** | 0.0935 | 1.4 | Up |
| N-Acetyl-glucosamine-1-phosphate | 4899.000 (227.699) | 3583.250 (789.917) | **0.0408** | 0.0935 | 1.37 | Up |
| Phenylalanine | 103881.667 (14008.093) | 74389.750 (14185.613) | **0.041** | 0.0935 | 1.4 | Up |
| Pantothenate | 148287.000 (9748.364) | 100054.750 (29124.841) | **0.0428** | 0.0935 | 1.48 | Up |
| Valine | 752777.667 (65873.229) | 555834.000 (113197.701) | **0.0451** | 0.0935 | 1.35 | Up |
| Leucine/  Isoleucine | 407124.667 (38755.665) | 292803.000 (67065.050) | **0.0479** | 0.0935 | 1.39 | Up |
| UDP-D-glucuronate | 3466.000 (214.846) | 2546.250 (573.455) | **0.0487** | 0.0935 | 1.36 | Up |
| Taurine | 1725785.000 (241923.812) | 1213468.250 (280664.408) | 0.053 | 0.0935 | 1.42 | Up |
| Creatine | 18059.667 (2188.864) | 12030.500 (3805.670) | 0.0598 | 0.095 | 1.5 | nd |
| Tryptophan | 19716.000 (3854.468) | 14513.000 (1994.287) | 0.0647 | 0.0998 | 1.36 | nd |
| Lysine | 33733.333 (1909.989) | 25062.000 (6436.567) | 0.0778 | 0.1167 | 1.35 | nd |
| Glycerol-3-phosphate | 14805.000 (3162.049) | 9816.500 (3550.972) | 0.1128 | 0.1582 | 1.51 | nd |
| Deoxyuridine | 94553.000 (22392.856) | 66379.000 (30326.955) | 0.2365 | 0.3114 | 1.42 | nd |
| Glutathione-oxidized | 70274.000 (10007.490) | 55261.750 (28271.633) | 0.428 | 0.5453 | 1.27 | nd |
| UMP | 13878.333 (7208.172) | 9788.250 (5835.500) | 0.4422 | 0.5453 | 1.42 | nd |
| Phosphocreatine | 2004.000 (1455.589) | 1207.250 (329.961) | 0.4443 | 0.5453 | 1.66 | nd |
| Acetylphosphate | 1661.000 (161.583) | 1425.750 (477.655) | 0.4587 | 0.5505 | 1.17 | nd |
| Palmitic acid | 3775.000 (126.787) | 5274.000 (4218.987) | 0.5287 | 0.6075 | -1.4 | nd |
| CMP | 3525.000 (1643.201) | 3052.750 (1808.175) | 0.7374 | 0.8296 | 1.15 | nd |
| GMP | 3282.333 (2640.008) | 2727.750 (2640.481) | 0.7943 | 0.8754 | 1.2 | nd |
| Stearic acid | 2307.000 (349.326) | 2202.750 (749.444) | 0.8348 | 0.9015 | 1.05 | nd |
| UDP | 11223.667 (9037.621) | 10323.500 (8061.487) | 0.8947 | 0.9229 | 1.09 | nd |
| Homocysteic acid | 218.000 (29.462) | 212.500 (70.718) | 0.9058 | 0.9229 | 1.03 | nd |
| GDP | 4383.667 (2272.162) | 4319.500 (3034.690) | 0.9769 | 0.9768 | 1.01 | nd |
| Aspartate | 156721.667 (7660.663) | 99360.000 (62607.933) | 0.0571 (W) | 0.0935 | 1.58 | nd |
| Histidine | 92466.667 (6472.957) | 65907.000 (12669.781) | 0.0571 (W) | 0.0935 | 1.4 | nd |
| Serine | 42163.667 (5503.506) | 30740.250 (6310.282) | 0.0571 (W) | 0.0935 | 1.37 | nd |
| Threonine | 140959.667 (10109.626) | 99782.750 (17344.957) | 0.0571 (W) | 0.0935 | 1.41 | nd |
| UDP-N-acetyl-D-glucosamine | 78504.667 (3569.184) | 53951.500 (11815.670) | 0.0571 (W) | 0.0935 | 1.46 | nd |
| Citrulline | 2375.667 (363.451) | 1805.250 (366.703) | 0.1143 (W) | 0.1582 | 1.32 | nd |
| Hexose glucose etc. | 78649.333 (8943.522) | 54761.000 (14553.406) | 0.1143 (W) | 0.1582 | 1.44 | nd |
| Inosine | 38546.000 (40457.692) | 14970.250 (17233.861) | 0.2286 (W) | 0.3086 | 2.57 | nd |
| Oleic acid | 1087.000 (136.231) | 3600.250 (5092.449) | 0.8571 (W) | 0.9076 | -3.31 | nd |

Statistical analysis was performed by Student’s two-tailed t-test, unless otherwise noted, i.e.,

(W) means calculation done using Wilcoxon Mann Whitney Test.

nd = not determined with confidence
