# Supplementary material for: Glycolysis Is an Intrinsic Factor for Optimal Replication of a Norovirus
Source: mBio. 2019 Mar 12;10(2):e02175-18. doi: 10.1128/mBio.02175-18 (PMC6414699; doi:10.1128/mBio.02175-18)

Supplemental Figure S1. Cell viability data for RAW cells treated with compounds used in this manuscript.

Cell viability assay (Resazurin reagent) on RAW cells treated with (A) 2DG for 24h; (B) 6AN for 24 h; and MK2206 for (C) 8h and (D) 24h. (E) Cell viability assay (WST-1 reagent) on Caco-2 cells with 2DG for 24h.

S1.

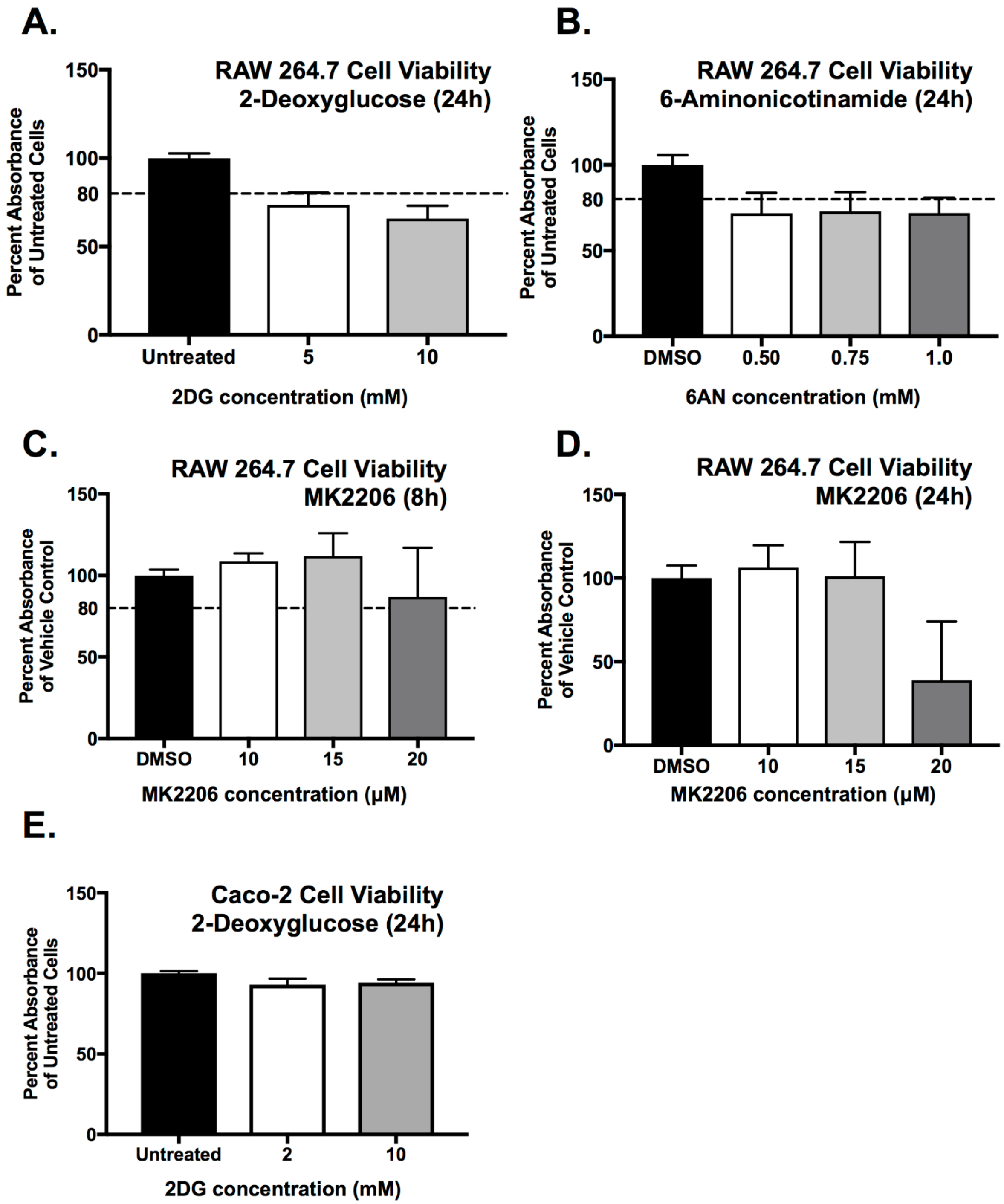

Supplement: FIG S1 [file mBio.02175-18-sf001.pdf]
