# Supplementary material for: Glycolysis Is an Intrinsic Factor for Optimal Replication of a Norovirus
Source: mBio. 2019 Mar 12;10(2):e02175-18. doi: 10.1128/mBio.02175-18 (PMC6414699; doi:10.1128/mBio.02175-18)
Supplement: TABLE S3 [file mBio.02175-18-st003.docx]

**Supplemental Table 3. Descriptive Statistics of Seahorse Real-Time ATP Rate Assay for RAW 264.7 cells infected with MNV for 8 hours.**

|  | **Glycolysis-ATP (pmol/min)** | | | **Mitochondrial-ATP (pmol/min)** | | |
| --- | --- | --- | --- | --- | --- | --- |
|  | **Mean^1^** | **SD^3^** | **N^3^** | **Mean^2^** | **SD^3^** | **N^3^** |
| Mock | 176.78 | 32.85 | 15 | 469.004 | 89.58 | 15 |
| MNV | 290.45 | 66.73 | 18 | 559.27 | 121.56 | 18 |
| Mock+2DG | 93.12 | 25.86 | 18 | 395.25 | 54.24 | 18 |
| MNV+2DG | 87.33 | 47.71 | 15 | 373.19 | 167.54 | 15 |

^1^Mean of Glycolysis-ATP in mock versus MNV infected is statistically significant with *P*=0.0029 (2-Way ANOVA with Tukey’s multiple comparisons test)

^2^Mean of Mitochondrial-ATP in mock versus MNV infected is significantly different with *P*=0.0188 (2-Way ANOVA with Tukey’s multiple comparisons test)

^3^N = total number of wells assessed and compiled from three separate biological experiments and SD = Standard Deviation
