# Supplementary material for: Glycolysis Is an Intrinsic Factor for Optimal Replication of a Norovirus
Source: mBio. 2019 Mar 12;10(2):e02175-18. doi: 10.1128/mBio.02175-18 (PMC6414699; doi:10.1128/mBio.02175-18)

**Supplemental Figure S2. 2DG inhibits viral production in RAW cells after transfection with MNV vRNA.**

(A) Transfection of vRNA into RAW cells and treatment with 10 mM 2DG results in about 1 $\log_{10}$ less plaque forming units compared to untreated cells after 24 hours (two experiments combined). Strand-specific RT-qPCR of (B) plus (+) and (C) minus (-) MNV vRNA strands from BMDM infected with MNV-1 for 4, 8 and 12 hours with and without 2DG treatment (10 mM). Mann-Whitney test used for (A). PFU = plaque forming units. Two-way ANOVA with Sidak's multiple comparisons test used for (B) and (C). ***P*<0.01; *****P*<0.0001; ns = not significant.

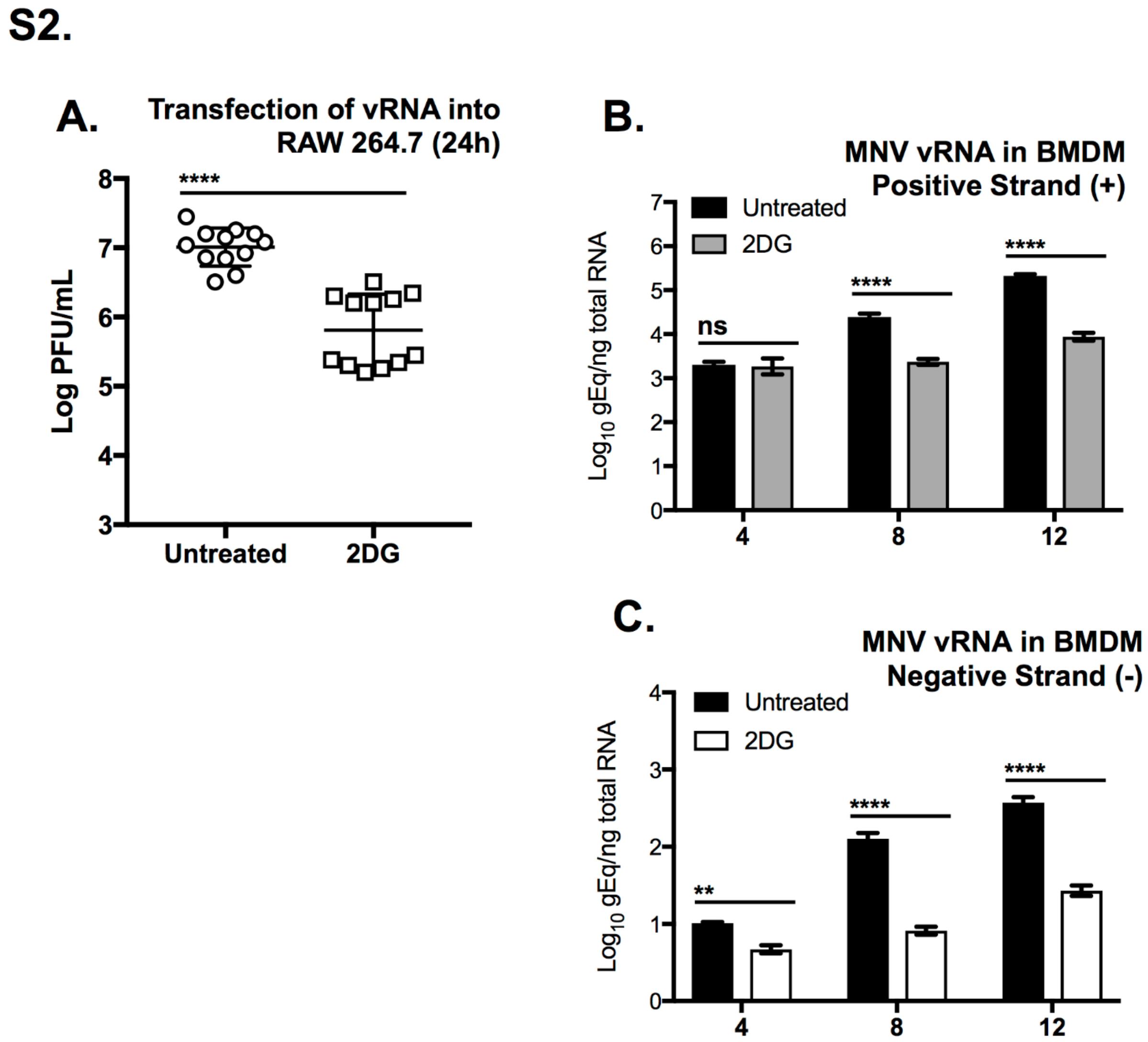

Supplement: FIG S2 [file mBio.02175-18-sf002.pdf]
