# Supplementary material for: Glycolysis Is an Intrinsic Factor for Optimal Replication of a Norovirus
Source: mBio. 2019 Mar 12;10(2):e02175-18. doi: 10.1128/mBio.02175-18 (PMC6414699; doi:10.1128/mBio.02175-18)

Supplemental Figure S3. Densitometry analysis of Western Blots of RAW cells infected with MNV for 2 hours. Densitometry measurements of phospho-AMPK(Thr172) and phospho-Akt(Ser473) in RAW cells after two hours infection. Protein normalized to actin and compared to mock infected samples.

S3.

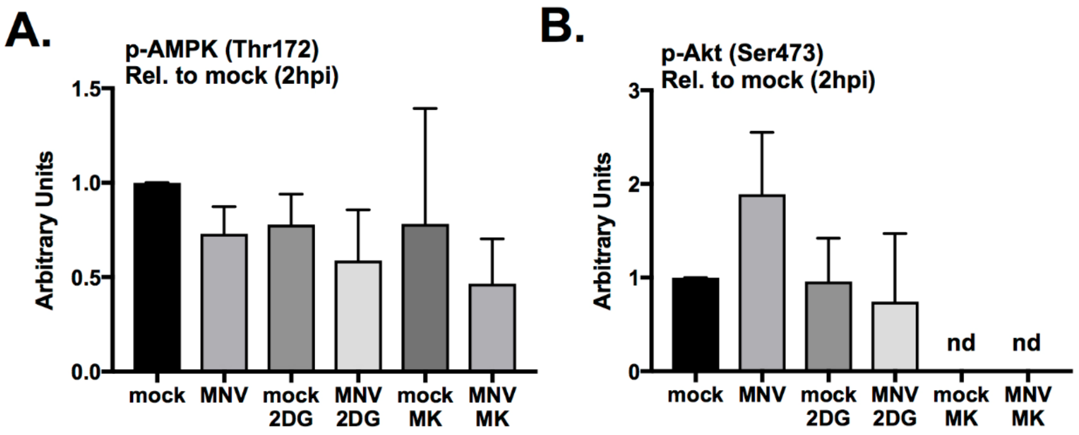

Supplement: FIG S3 [file mBio.02175-18-sf003.pdf]
