# Supplementary material for: Glycolysis Is an Intrinsic Factor for Optimal Replication of a Norovirus
Source: mBio. 2019 Mar 12;10(2):e02175-18. doi: 10.1128/mBio.02175-18 (PMC6414699; doi:10.1128/mBio.02175-18)

Supplemental Figure S4. 2DG treatment causes a strong increase in Akt phosphorylation in RAW cells after 12 hours. Western blot of Akt and phospho-Akt (Ser473) in RAW cells infected with MNV (MOI=5) or mock infected and treated with 10 mM 2DG or 15 μM MK2206. beta-Actin corresponds to top and bottom membranes. Numbers represent densitometry measurement of protein normalized to actin and compared to mock infected cells. nd = not detected.

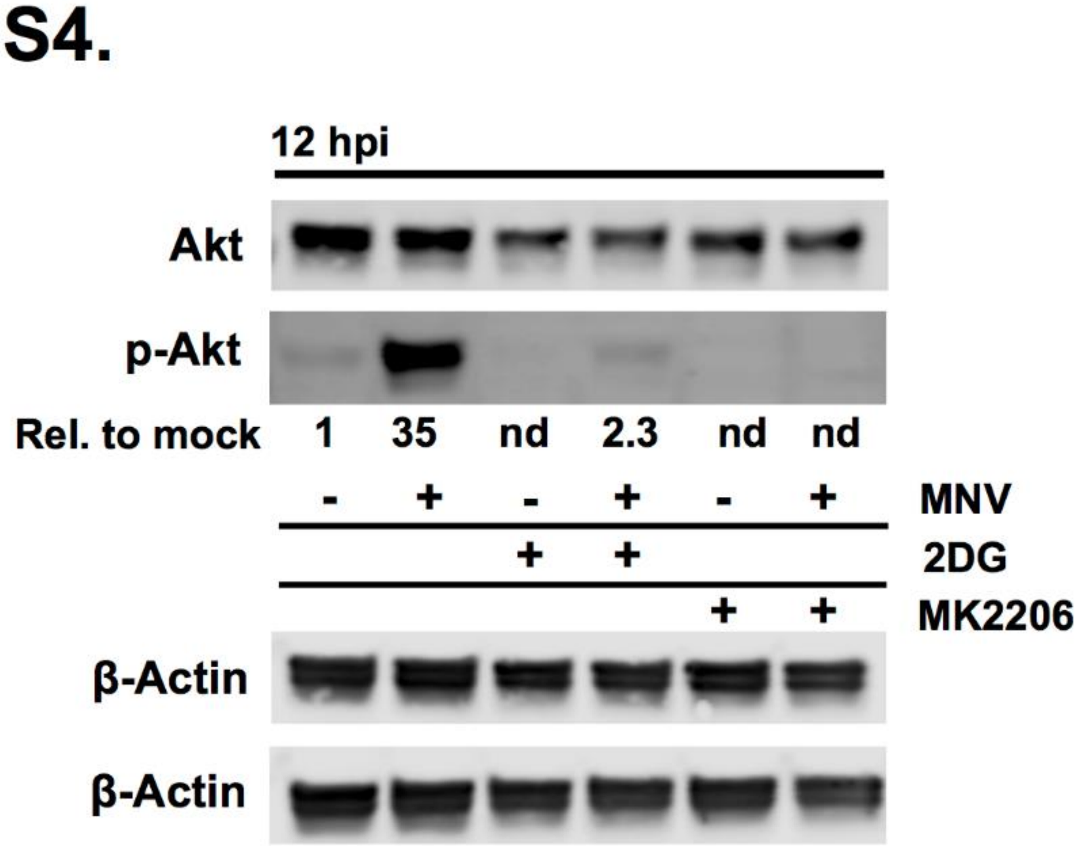

Supplement: FIG S4 [file mBio.02175-18-sf004.pdf]
