# Supplementary material for: Glycolysis Is an Intrinsic Factor for Optimal Replication of a Norovirus
Source: mBio. 2019 Mar 12;10(2):e02175-18. doi: 10.1128/mBio.02175-18 (PMC6414699; doi:10.1128/mBio.02175-18)

Supplemental Figure S5. Analysis of General Reactive Oxygen Species (ROS) in RAW 264.7 cells. RAW 264.7 cells were pre-treated for 30 minutes with 2DG or PMA and stained with the general ROS reactive dye H2DCFDA. 2DG samples were then grown with 2DG for an additional 30 minutes. Fluorescence was measured by flow cytometry. VC = vehicle control, DMSO at v/v match to experimental compounds.

S5.

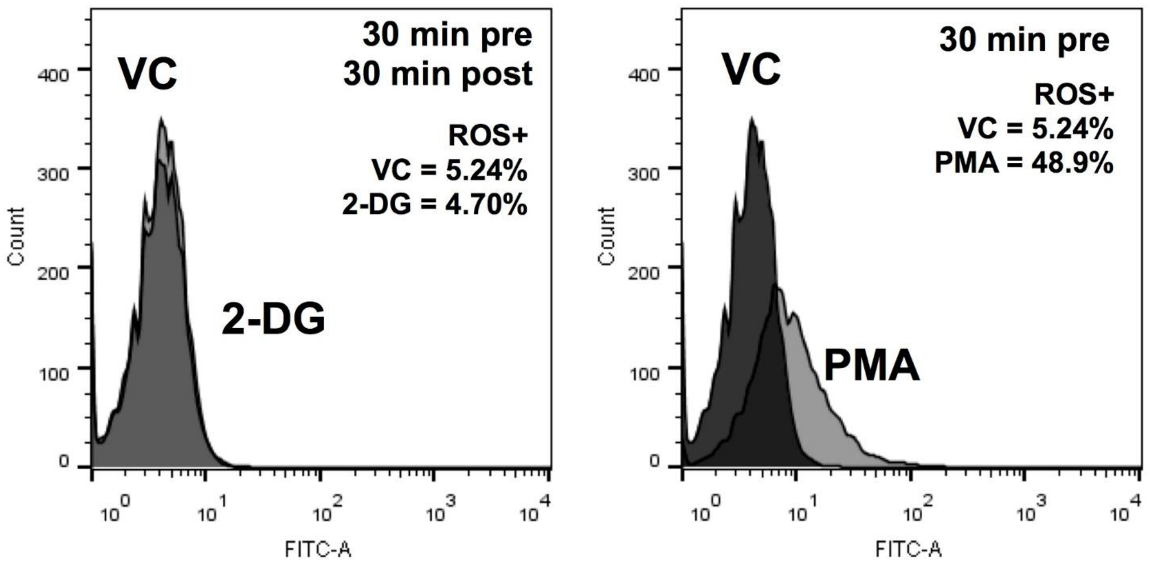

Supplement: FIG S5 [file mBio.02175-18-sf005.pdf]
