# Supplementary material for: Glycolysis Is an Intrinsic Factor for Optimal Replication of a Norovirus
Source: mBio. 2019 Mar 12;10(2):e02175-18. doi: 10.1128/mBio.02175-18 (PMC6414699; doi:10.1128/mBio.02175-18)

Supplemental Figure S6. 2DG treatment does not cause a significant decrease in TNFα during MNV infection.

Results of ELISA measurement of TNFα in cell lysates from RAW 264.7 cells infected with MNV (MOI=5) for 8 hours and treated with 2DG as indicated. Kruskal-Wallis test with Dunn's multiple comparisons post-test: ***P*<0.01, ns = not significant.

S6.

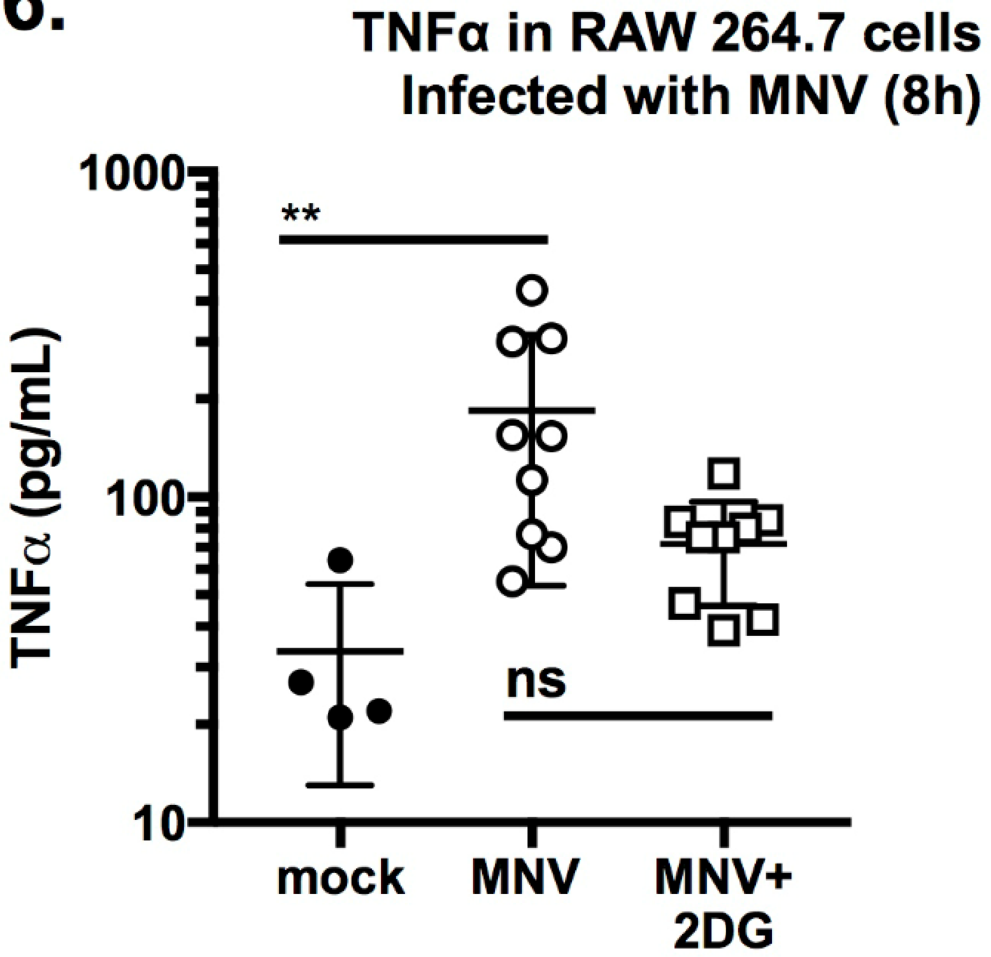

Supplement: FIG S6 [file mBio.02175-18-sf006.pdf]
