# Supplementary material for: Glycolysis Is an Intrinsic Factor for Optimal Replication of a Norovirus
Source: mBio. 2019 Mar 12;10(2):e02175-18. doi: 10.1128/mBio.02175-18 (PMC6414699; doi:10.1128/mBio.02175-18)
Supplement: TEXT S1 [file mBio.02175-18-s001.docx]

**Supplemental Text S1:**

**Detailed Materials and Methods**

**Compounds and reagents:** 2-Deoxyglucose (2DG) (Sigma D8375) was solubilized fresh per each experiment in cell culture medium to 100 mM and added to experimental medium at a final concentration of 10 mM (or as indicated). MK2206 2HCL (Selleckchem S1078) was solubilized in DMSO at 10 mM and used at a final concentration of 15 μM. 6-Aminonicotinamide (6AN) (Cayman 10009315) was solubilized in DMSO at 500 mM and used at 500 or 750 μM. Oligomycin A (Cayman 11342) was solubilized in DMSO at 5 mM and used at 1 μM.

**Cell culture and virus strains:** The RAW 264.7 macrophage-like cell line (referred herein as RAW) (ATCC TIB-71) and Caco-2 cells were maintained in DMEM-10 medium (Gibco DMEM medium #11995-065 with 4.5 g/L D-Glucose and 110 mg/L Sodium Pyruvate, 10% Fetal Bovine Serum [HyClone] 1% Penicillin/Streptomycin [Gibco 15140-122], 1% HEPES buffer [Gibco 1M 15630-080], 1% Non-Essential Amino Acids [Gibco 100X 11140-050] and 1% L-Glutamine [Gibco 200mM 25030-081]) in treated tissue culture flasks at 37°C/5% CO_2_. All experiments used RAW cells below passage 14. Primary bone marrow derived macrophages (BMDM) were differentiated from male Balb/C femur and tibia bone marrow in 20% L929 medium (Gibco DMEM medium, 20% FBS [HyClone], L9 supernatant, 1% L-Glutamine, 1% Sodium Pyruvate, 0.25 mL β-mercaptoethanol/L and 2% Penicillin/Streptomycin) and frozen in liquid nitrogen until use. All experiments using primary cells were performed with 10% L929 working medium (same as DMEM-10 above but with 10% L929 supernatant). The plaque purified MNV-1 clone (GV/MNV1/2002/USA) MNV-1.CW3 (1) (referred herein as MNV-1) was used at passage 6 in all experiments.

**Virus infections, virus transfection and plaque assay:** All MNV infections were done in the RAW 264.7 cell line, Balb/c primary bone marrow-derived macrophages (BMDM from male mice) or BMDM from WT and IFNAR1-knockout cells on a C57Bl6 background. Cells were grown in 12-well tissue culture plates seeded at 5 X 10^5 cells/well. At the time of infection, medium was replaced with 500 μL of medium containing MNV-1 at indicated MOI, plates were rocked for 1 hour on ice. Then, cells were washed 3X with cold DPBS++ (+Calcium and +Magnesium Chloride – Gibco 14040), fresh medium was added, and cells were incubated for indicated times. Cells were then frozen at -80°C, freeze-thawed two times, before lysates were analyzed by plaque assay as described (2). For transfection of viral RNA into RAW cells, RAW cells were plated in 12-well plates at 5 X10^5 cells/well in medium containing no antibiotics. Cells were transfected using 2 μL of Dharmacon Dharmafect 4 per reaction in Serum-free OptiMem medium (Gibco 11058) (per manufacturer’s protocol) using 1 μg of total RNA collected from previously MNV-infected RAW cells (see RNA protocol). Cells were incubated for 12 or 24 hours with and without 10 mM 2DG, frozen at -80°C, freeze-thawed twice and lysates were tested for infectious viral particles via plaque assay (2). Primary cell infections were done the same as RAW infections except in medium containing 10% L929 supernatant. Infections using Ribose included Ribose dissolved fresh (on the day of the experiment) into tissue-culture medium and filter sterilized through a 0.2 μM filter flask. Nucleosides were acquired from Selleckchem except Thymidine (catalog numbers: Adenosine S1647; Cytidine S2053; Guanosine S2439; Thymidine Sigma T1895; and Uridine S2029) and were solubilized in DMSO prior to being diluted into medium to 50 μM. Vehicle controls were performed using DMSO in a v/v match to the volume added with test reagents. For human astrovirus infection, Caco-2 (10^5) cells were seeded in 48-well tissue culture plates overnight. Cells were infected with astrovirus VA1 (MOI=1) for 1 hour at 37°C. Media was removed and cells were washed 2X with PBS. Media containing increasing concentrations of 2DG was then added to the cells and incubated at 37°C. At days 1 and 3 post infection, RNA was extracted from the infected cells using Direct-zol RNA miniprep plus (Zymo Research) according to the manufacturer's protocol. Virus titer was determined by RT-qPCR as described (3).

**Cell viability assay:** Cell viability was tested using Resazurin reagent (Biotium 30025-1). Briefly, cells were plated in 96-well flat bottom tissue culture plates at 35,000 (RAW) or 40,000 (BMDM) cells/well and incubated overnight at 37°C/5% CO2. Medium was removed and replaced with 100 μL of fresh medium with reagent of interest at indicated concentrations and times. Resazurin (10 μL/well) was added and plates were incubated at 37°C/5% CO2 for at least 3 hours and then read on a BioTek plate reader at absorbances 570 and 600 nm. Then A600 was subtracted from A570 and experimental samples were compared to untreated or vehicle-treated controls to calculate percent of control absorbance. WST-1 assay was used per the manufacturer’s directions for Caco-2 cells.

**RNA extraction and RT-qPCR:** Experiments using RNA were performed as indicated above and at time of RNA extraction, cells were washed 1X with cold DPBS++ and then 500 μL or 1 mL of Trizol (Ambion 15596018) or Zymo Research TriReagent (R2050-1) was added. Extraction was performed per manufacturer’s directions using Chloroform extraction (Trizol) or the Zymo Research Direct-zol RNA MiniPrep Plus (R2072), quantitated on the NanoDrop spectrophotometer, and then used for One-Step TaqMan Assay (MNV).

**Strand-specific RT-qPCR:** Strand-specific RT-qPCR for MNV was performed as previously described (4). Briefly, after infection as described above, RNA was extracted as described, and RNA concentration was normalized to 100 ng/μl. Strand-specific standard RNAs were synthesized by PCR, gel purified and serially diluted from 1 × 10 copies/μl to 10 copies/μl. Two μl of normalized total RNA or standard RNA was used per reverse transcription reaction (RT) with strand-specific RT primers. After the RT reactions, the cDNA was diluted 1:10 and 5 μl was used for strand-specific qPCR. For each sample, one RT reaction was performed with duplicated qPCR reactions. The results were calculated based on the standard curves and extrapolated to genome equivalent (gEq) per ng total RNA and data represent three independent biological replicates.

**Protein extraction, SDS-PAGE and immunoblotting:**  Experiments were performed as described above in 12-well or 6-well tissue culture plates and at time of harvest, cells were washed 2X with cold DPBS++ and RIPA buffer (Pierce 89900) with PhosSTOP (Roche 04906845001), and cOmplete EDTA-free protease inhibitor cocktail (Roche 11873580001) was added to wells. Cells were scraped, moved to sterile centrifuge tubes and incubated on ice for 10-15 minutes. Cells were then spun at 4^o^C at 14,000 x g for 15 minutes. Lysates were moved to fresh tubes, and Laemmli buffer with β-mercapto-ethanol added at 3:1 lysate to buffer before freezing the sample until analysis. SDS-PAGE was performed with BioRad 4-20% Mini-Protean TGX gels (BioRad 456-1096) per standard SDS-PAGE procedures. Gels were transferred to Immobilon-FL transfer membranes (IPFL00010 Pore size 0.45 μm) using a Semi-Dry transfer at 10V for 35-60 minutes. Membranes were blocked in PBS+0.05% Tween + 5% BSA for one hour at room temp, then primary antibodies were added in the same buffer and membranes were rocked at 4°C overnight. Membranes were washed 4X with 1X PBS, then secondary LI-COR fluorescent antibodies were added for 1.5 hours at room temp and then visualized on the LI-COR Odyssey Imager. Western blots were quantified by densitometry using ImageJ and normalizing bands to β-actin. Cell Signaling Antibodies used: #3700 β**–**Actin (8H10D10) Mouse mAb; #8457 β-Actin (13E5) Rabbit mAb; #4685 Akt (pan) (11E7) Rabbit mAb; #4058 Phospho-Akt (Ser473) (193H12) Rabbit mAb; #2793 AMPKα (F6) Mouse mAB; #4188 Phospho-AMPKα (Thr172) (D79.5E) Rabbit mAb. The rabbit polyclonal anti-MNV-1 ProPol antibody was a kind gift of Dr. Kim Green (NIH, NIAID, USA) (5). The rabbit polyclonal anti-MNV-1 capsid antibody was described previously (6).

**Metabolomics assay:** For metabolomics experiments, RAW 264.7 cells were plated in 10 cm Tissue Culture plates with 6.5 X 10^6 cells and grown at 37°C/5% CO2 overnight. In the morning, fresh culture medium (DMEM-10) with MNV-1 at MOI = 5 or medium containing RAW cell lysate in v/v ratio of virus medium (mock infection) was added to plates and they were rocked on ice for one hour. Cells were washed 3X with cold DPBS++ and then incubated at 37°C/5% CO2 for 7.5 hours. Cells were then washed 2X with 150 mM Ammonium Acetate and immediately quenched with liquid nitrogen then placed on a bed of dry ice. Plates were then moved to the -80°C freezer and wrapped in foil until transport to the Michigan Regional Comprehensive Metabolomics Resource Core (MRC^2^) at the University of Michigan for analysis by Mass Spectrometry. Sample preparation at the MRC^2^ was as follows: Cell culture plates were removed from -80 °C storage and maintained on wet ice throughout the processing steps. To each 10 cm plate, 1.0 mL of a mixture of methanol, chloroform and water (8:1:1) containing isotope labeled internal standards was added. Plates were gently agitated to release cells, and then scraped to homogenize cells, and the cell mixture quantitatively transferred to a microtube. Microtubes were vortexed, and allowed to incubate at 4°C for 10 minutes to complete metabolite extraction. Samples we vortexed a second time, and then centrifuged at 14,000 RPM for 10 min at 4 °C. 100 µL of the extraction solvent was transferred to an autosampler vial for LC-MS analysis. 10 µL of each sample was removed and pooled in a separate autosampler vial for quality control purposes. LC-MS analysis at the MRC^2^ was as follows: Glycolysis/TCA/PPP analysis was performed on an Agilent system consisting of a 1290 UPLC coupled with a 6520 Quadrupole-Time-of-flight (QTOF) mass spectrometer (Agilent Technologies, Santa Clara, CA.) Metabolites were separated on a 150x1mm Luna NH_2_ Hilic column (Phenomenex, Torrance, CA) using 10 mM ammonium acetate in water, adjusted to pH 9.9 with ammonium hydroxide, as mobile phase A, and acetonitrile as mobile phase B. The flow rate was 0.075 mL/min and the gradient was linear from 20% to 100% A over 15 mins, followed by isocratic elution at 100% A for 5 minutes. The system was returned to starting conditions (20% A) and held there for 10 minutes to allow for column re-equilibration before injecting another sample. The mass spectrometer was operated in ESI- mode according to previously published conditions (7). Data were analyzed in Metaboanalyst using an FDR of 0.05 for t-test calculations.

**Lactate assay:** MNV infections were performed as described above and supernatants were assessed for lactate using the Cayman Chemical Glycolysis Cell-Based Assay Kit (600450) per the manufacturer’s protocol.

**ELISA:** Cytokine levels were determined at the University of Michigan Rogel Cancer Center Immunological Monitoring Core by ELISA (Duosets, R&D Systems, Minneapolis, MN) using the manufacturer’s recommended protocol modified to an overnight sample incubation. Assays were developed using TMBX substrate (Surmodics, Eden Prairie, MN) and stopped with an equal volume of 0.4% NaF. Results represent combined results from three independent experiments.

**ROS measurements.** Macrophages were pre-treated with 10 mM 2DG or 1 micromolar Phorbol 12-myristate 13-acetate (PMA – positive control) for 30 minutes. Staining was done with 5 μM CM-H2DCFDA (general ROS indicator) in HBSS buffer in the dark for 30 minutes. Cells were then washed with warm culture medium with 10 mM 2DG and incubated for 30 minutes at 37°C/5% CO2. Cells were then washed 2X with warm DPBS++, lifted from culture plates, and measured on the FACS Canto flow cytometers (BD Biosciences) on FITC-A with 20,000 events on medium flow. Data were analyzed using FlowJo software.

**Agilent Seahorse XF Real-Time ATP Rate Assay:** The XF ATP Rate Assay (Agilent 103592-100) was used per the manufacturer’s instructions as follows. RAW 264.7 cells were seeded in 96 well Seahorse XF96 Cell Culture Microplates at 2 X 10^4^ and 3 X 10^4^ cells/well in 80 μL of normal tissue culture medium (see Cell Culture above). Cells were allowed to recover overnight for 16-17 hours at 37°C/5% CO_2_. In the morning, medium was changed for 100 μL of medium with mock (RAW cell lysate) or MNV-1 at MOI=5 and rocked on ice for one hour to infect. Cells were washed 1X with warm medium and incubated for 7 hours at 37°C/5% CO_2_. Cells were then washed 1X with 200 μL warm ATP Assay Medium prepared per protocol (Agilent XF DMEM Medium pH 7.4 (103757-100); 10 mM XF Glucose; 1 mM XF Sodium Pyruvate; 2 mM XF L-Glutamine) and then fresh ATP Assay medium was added for a final well volume of 180 μL. Cells were incubated at 37°C (non-CO_2_) for 45 minutes, medium was removed and replaced with 200 μL fresh ATP Assay medium, then put on the Seahorse XFe96 Extracellular Flux Analyzer in the University of Michigan Pathology department. Agilent XFe96 Extracellular Flux Assay cartridges were hydrated overnight in pure water, and in the morning, water was changed for Seahorse Calibrant and incubated at 37°C (non-CO_2_). Data were analyzed in the Agilent ATP Assay Report Generator and statistics were analyzed in Prism 7.0.

**Statistical Analysis:** Metabolomics data were analyzed in Metaboanalyst 4.0. For all other experiments, data were analyzed in Prism7 using the tests as indicated in Figure legends.

**Supplemental References**

1. Thackray LB, Wobus CE, Chachu KA, Liu B, Alegre ER, Henderson KS, Kelley ST, Virgin HWt. 2007. Murine noroviruses comprising a single genogroup exhibit biological diversity despite limited sequence divergence. J Virol 81:10460-73.

2. Gonzalez-Hernandez MB, Bragazzi Cunha J, Wobus CE. 2012. Plaque assay for murine norovirus. J Vis Exp doi:10.3791/4297:e4297.

3. Janowski AB, Bauer IK, Holtz LR, Wang D. 2017. Propagation of astrovirus VA1, a neurotropic human astrovirus, in cell culture. J Virol doi:10.1128/JVI.00740-17.

4. Vashist S, Urena L, Goodfellow I. 2012. Development of a strand specific real-time RT-qPCR assay for the detection and quantitation of murine norovirus RNA. J Virol Methods 184:69-76.

5. Sosnovtsev SV, Belliot G, Chang KO, Prikhodko VG, Thackray LB, Wobus CE, Karst SM, Virgin HW, Green KY. 2006. Cleavage map and proteolytic processing of the murine norovirus nonstructural polyprotein in infected cells. J Virol 80:7816-31.

6. Wobus CE, Karst SM, Thackray LB, Chang KO, Sosnovtsev SV, Belliot G, Krug A, Mackenzie JM, Green KY, Virgin HW. 2004. Replication of Norovirus in cell culture reveals a tropism for dendritic cells and macrophages. PLoS Biol 2:e432.

7. Lorenz MA, Burant CF, Kennedy RT. 2011. Reducing time and increasing sensitivity in sample preparation for adherent mammalian cell metabolomics. Anal Chem 83:3406-14.
